# Multi-Organ RNA Virome Profiling of Edible Rodents Reveals Potential Zoonotic Viral Exposure at the Wildlife–Livestock–Human Interface in Southwest China

**DOI:** 10.3390/pathogens15050558

**Published:** 2026-05-21

**Authors:** Dijun Chen, Jingzhu Zhou, Qing Ma, Xuexue Kong, Shijun Li, Qiyong Liu, Wenqin Liang

**Affiliations:** 1 Key Laboratory of Environmental Pollution Monitoring and Disease Control, Ministry of Education, School of Public Health, Guizhou Medical University, Guiyang 561113, China; 13320305882@189.cn (D.C.); 15117332904@163.com (X.K.); 2 Key Laboratory of Microbiome and Infectious Disease Prevention and Control, Department of Vector Surveillance Section of Guizhou Center for Disease Control and Prevention, Guiyang 550004, China; gtz_gfk@163.com (J.Z.); mq2001069@163.com (Q.M.); 3 State Key Laboratory of Infectious Disease Prevention and Control, Collaborative Innovation Center for Diagnosis and Treatment of Infectious Diseases, National Institute for Communicable Disease Control and Prevention, Chinese Center for Disease Control and Prevention, Beijing 102206, China

**Keywords:** edible rodents, RNA virome, Seoul virus, porcine Rotavirus A, One Health, wildlife–livestock interface, zoonotic risk

## Abstract

The consumption of wild rodents in certain regions of Southwest China creates a potential interface for zoonotic pathogen exposure, yet the virome composition of edible rodents remains insufficiently characterized. In this study, we performed multi-organ RNA metatranscriptomic analysis of three commonly consumed rodent species (*Niviventer andersoni*, *Berylmys bowersi*, and *Rattus losea*) collected from Guizhou Province, analyzing five visceral organs per species. A total of 1198 viral contigs spanning 37 viral families were identified, revealing diverse viral communities across host species and tissues, with host identity emerging as a key factor shaping virome structure. Sequences related to Seoul virus were detected in the lungs of *R. losea*, showing high similarity to previously reported strains, and sequences closely related to porcine Rotavirus A were identified in the lung samples of *N. andersoni*, indicating a close phylogenetic relationship with livestock-associated viruses. While these findings do not confirm active infection or transmission, they may reflect potential environmental exposure or ecological links at the wildlife–livestock interface. Overall, this study provides a baseline characterization of the multi-organ virome of edible rodents and highlights the importance of integrated surveillance and risk assessment within a One Health framework.

## 1. Introduction

Rodents represent the most speciose and globally distributed mammalian order, playing essential roles in ecosystem maintenance while serving as primary reservoirs for over 60 known zoonotic pathogens [1]. High-consequence agents, such as *Orthohantaviruses* and *Lassa mammarenavirus*, pose persistent threats to global public health [2,3]. Zoonotic spillover events are frequently driven by anthropogenic encroachment into natural habitats and specific dietary behaviors. The consumption of rodents as a supplementary protein source is a historically entrenched practice across parts of Southeast Asia, Africa, and South America [4,5,6].

A similar tradition persists in the Qiandongnan Miao and Dong Autonomous Prefecture in Guizhou, Southwest China, yet this custom carries significant biosecurity risks. Residents frequently slaughter wild rodents manually without protective equipment, leading to cross-contamination between viscera and muscle tissue. Furthermore, the consumption of raw or undercooked viscera—and even the use of intestinal contents for seasoning—is not uncommon [7]. These practices create multiple high-risk exposure pathways, including aerosol inhalation, direct mucosal contact, and ingestion. Despite these risks, prior virological surveillance has predominantly focused on fecal samples. As a result, the viral landscapes of high-risk parenchymal organs—such as the lungs, liver, and kidneys—remain largely unexplored, hindering a comprehensive assessment of public health risks.

In virome studies, alpha diversity describes within-sample viral diversity, with Chao1 estimating richness and Simpson reflecting both richness and dominance [8]. Beta diversity evaluates differences in viral community composition among samples; here, Bray–Curtis-based PCoA was used to visualize variation associated with host species and tissue type [9].

Next-generation sequencing, particularly metatranscriptomics, provides an unbiased approach for characterizing host viromes and identifying divergent pathogens [10,11,12]. In this study, metatranscriptomic sequencing was used to characterize RNA viral diversity because it detects actively transcribed viral sequences and is well suited for identifying potentially active RNA viruses. Compared with total nucleic acid-based virome approaches, this method is more focused on RNA viruses and may underestimate DNA viruses that are not actively transcribed; nevertheless, it is appropriate for the aim of this study [13].

To characterize these risks, this study targeted three predominant edible rodent species in the Qiandongnan region: *N. andersoni* (NA), *B. bowersi* (BB), and *R. losea* (RL). Rodents of the genus *Rattus*, including *R. losea*, are well-recognized reservoirs of zoonotic viruses, including hantaviruses responsible for hemorrhagic fever with renal syndrome (HFRS), as well as rat hepatitis E virus and rodent-associated coronaviruses with cross-species transmission potential [14,15,16].

Although direct virological evidence for *N. andersoni* (NA), *B. bowersi* (BB), and *R. losea* (RL) remains limited, phylogenetically and ecologically related rodent taxa in China and Southeast Asia have been shown to harbor hantaviruses and other RNA viruses, suggesting that these species may contribute to the maintenance and transmission of zoonotic pathogens in local ecosystems [17,18].

We performed deep metatranscriptomic sequencing on five distinct visceral organs to: (i) characterize the RNA viral communities across tissue types; (ii) evaluate the influence of host species and organ specificity on virome structure; (iii) identify key zoonotic agents and their evolutionary characteristics; and (iv) assess potential viral exposure and ecological links at the wildlife–livestock–human interface in the context of local dietary customs.

## 2. Materials and Methods

### 2.1. Sample Collection

**Figure 1:**
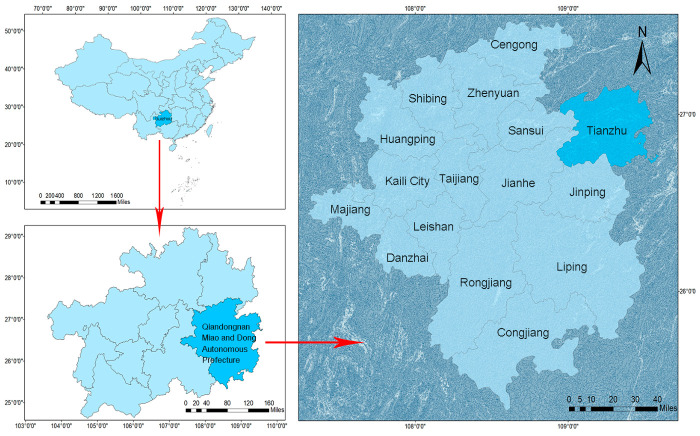
Geographic location of sampling sites in Tianzhu County, Qiandongnan Miao and Dong Autonomous Prefecture, Guizhou Province, Southwest China. The highlighted regions (blue) indicate the administrative boundaries of Guizhou Province and Qiandongnan Prefecture, with Tianzhu County shown in darker blue as the sampling area. Arrows indicate the hierarchical geographic relationship from the national to the local scale.

In October 2024, a total of 42 wild rodents were captured using the snap-trap method in Yuankou Town, Tianzhu County, Qiandongnan Miao and Dong Autonomous Prefecture, Guizhou Province (Figure 1). Based on morphological characteristics, 30 intact individuals representing three major edible species—*Niviventer andersoni* (NA, *n* = 10), *Berylmys bowersi* (BB, *n* = 10), and *Rattus losea* (RL, *n* = 10)—were selected for viromic analysis. The rodents were euthanized and necropsied under aseptic conditions. Five organs, namely, the lung (Lu), liver (Li), spleen (Sp), kidney (Ki), and intestine (In), were collected from each individual. To minimize individual variation and maximize sequencing depth, equal weights of the same organ tissue from ten individuals of the same species were pooled, resulting in 15 composite samples (3 species × 5 organ types). Pooling was performed to enhance viral detection sensitivity and sequencing depth, which is commonly applied in virome studies targeting low-abundance viral populations. All samples were immediately flash-frozen in liquid nitrogen and stored at −80 °C until further processing. Sample processing was conducted under strict sterile conditions, with laboratory precautions applied throughout RNA extraction and library preparation to minimize cross-contamination and nucleic acid degradation.

### 2.2. RNA Extraction, Library Construction, and Sequencing

Total RNA was extracted using the HiPure Universal RNA Mini Kit (Magen Biotech Co., Ltd., Guangzhou, China) according to the manufacturer’s instructions. RNA quality was assessed by 1% agarose gel electrophoresis, and concentration was measured using a Qubit 3.0 Fluorometer (Thermo Fisher Scientific, Waltham, MA, USA). RNA integrity was further evaluated using an Agilent 4200 Bioanalyzer (Agilent Technologies, Santa Clara, CA, USA). Sequencing libraries were constructed using the ALFA-SEQ RNA Library Prep Kit II (Magigene Biotechnology Co., Ltd., Guangzhou, China). Ribosomal RNA was removed to enrich non-ribosomal RNA fractions. The remaining RNA was subjected to fragmentation, followed by complementary DNA synthesis, end repair, adapter ligation, and polymerase chain reaction amplification. Library quality was assessed using a Qsep400 system (BiOptic Inc., New Taipei City, Taiwan, China). Paired-end sequencing (150 base pairs per read) was performed on an Illumina NovaSeq 6000 platform by Guangdong Magigene Biotechnology Co., Ltd. (Guangzhou, China).

### 2.3. Bioinformatics Pipeline and Viral Identification

**Figure 2:**
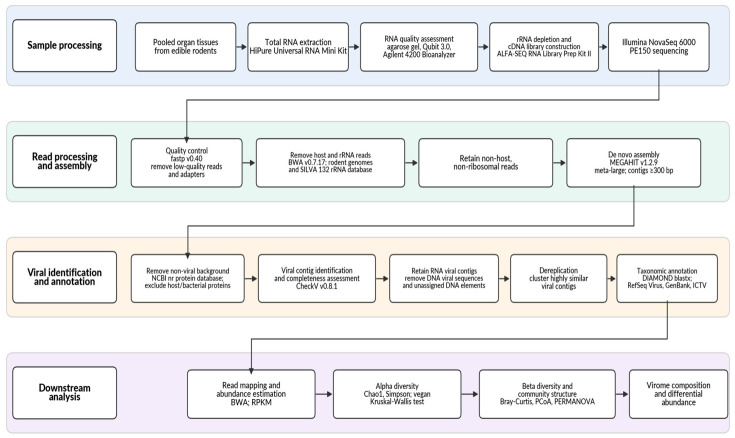
Workflow of RNA virome metatranscriptomic analysis. The pipeline includes RNA extraction, library construction, Illumina sequencing, quality control (fastp), removal of host and rRNA reads (BWA), de novo assembly (MEGAHIT), viral contig identification and quality assessment (CheckV), RNA virus filtering, sequence clustering, taxonomic annotation (DIAMOND against viral databases), and downstream analyses including abundance estimation, diversity analysis, and phylogenetic inference. Different colors indicate major analytical stages: sample processing (blue), read processing and assembly (green), viral identification and annotation (orange), and downstream analysis (purple). PE150, paired-end sequencing with 150 bp read length; RPKM, reads per kilobase per million mapped reads.

The overall workflow of sequencing, bioinformatic processing, viral identification, and downstream analyses is summarized in Figure 2. Raw sequencing reads were processed using fastp (version 0.40) [19] to remove low-quality reads and adapter sequences. High-quality clean reads were then aligned to rodent reference genomes and the Silva 132 ribosomal RNA database using the Burrows-Wheeler Aligner (version 0.7.17) [20]. Reads mapped to host genomes and ribosomal RNA sequences were removed, and the remaining non-host, non-ribosomal reads were retained for downstream analysis. De novo assembly of the filtered reads was performed using MEGAHIT (version 1.2.9) [21] with the “meta-large” preset and a minimum contig length of 300 base pairs. To further reduce non-viral background, assembled contigs were compared against the NCBI non-redundant protein database, and sequences with similarity to host or bacterial proteins were excluded. Viral contigs were identified and their completeness was evaluated using CheckV (version 0.8.1) [22]. To specifically characterize ribonucleic acid viruses, contigs classified as deoxyribonucleic acid viruses or unassigned deoxyribonucleic acid elements were removed. To obtain a non-redundant dataset, highly similar viral contigs were clustered based on sequence similarity. Taxonomic annotation was performed by aligning contigs against a customized viral protein database integrating NCBI RefSeq Virus, GenBank, and ICTV classifications using DIAMOND blastx [23,24,25]. Viral contigs were retained according to commonly applied metatranscriptomic criteria, including alignment identity and coverage.

### 2.4. Viral Abundance and Diversity Analysis

Clean reads were mapped back to the identified viral contigs using the Burrows-Wheeler Aligner, and viral abundance was calculated as reads per kilobase per million mapped reads. Alpha diversity indices, including Chao1 and Simpson indices, were calculated using the R package vegan (version 2.6-4), and differences among groups were evaluated using the Kruskal–Wallis test. Beta diversity was assessed using Bray–Curtis distances and visualized by principal coordinate analysis. Differences in community structure were tested using permutational multivariate analysis of variance (PERMANOVA), performed with 999 permutations.

### 2.5. Phylogenetic Analysis

For selected viruses of interest, including Seoul virus and Rotavirus A, RNA-dependent RNA polymerase sequences were extracted from assembled contigs. Reference sequences were retrieved from NCBI and selected to represent major phylogenetic lineages, geographically relevant strains, and closely related sequences identified through similarity searches. For SEOV and RVA, phylogenetic analyses were performed based on functionally conserved RNA-dependent RNA polymerase (RdRp) proteins. Specifically, the L segment-encoded RdRp was used for SEOV, while the VP1 protein, which serves as the RdRp in Rotavirus A, was used for RVA. These genes were selected because they are widely used markers for phylogenetic inference and genotype/species assignment. Only contigs longer than 300 bp covering conserved RdRp regions were retained, and their translated amino acid sequences were used for phylogenetic analysis. Although other genes or full genomes could provide higher resolution, fragmented assemblies and limited reference sequences restricted broader comparative analyses.

Multiple sequence alignments were performed on amino acid sequences using MUSCLE. Maximum likelihood (ML) phylogenetic trees were constructed in MEGA version 12 [26]. The best-fit amino acid substitution model was selected based on model testing implemented in MEGA. Only translated amino acid sequences with sufficient coverage of conserved RdRp regions were included in the final phylogenetic analysis. Node support was estimated using 1000 bootstrap replicates, with only bootstrap values greater than 70% displayed. Reference sequences were selected to represent major phylogenetic lineages and geographically relevant strains. This approach provides sufficient resolution to examine evolutionary relationships and potential zoonotic links for SEOV and RVA.

## 3. Results

### 3.1. Overview of Sequencing Data and Viral Assembly

**Figure 3:**
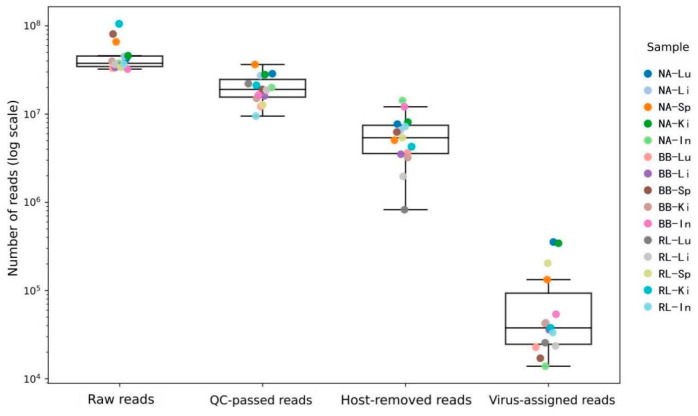
Stepwise data filtration dynamics and viral enrichment across rodent tissue transcriptomes. The boxplots illustrate the distribution of sequencing read counts (log scale) at four sequential bioinformatic processing stages: Raw reads, QC-passed reads, Host-removed reads, and Virus-assigned reads. Each dot represents a pooled tissue sample. Sample nomenclature follows the format [Species]–[Organ]. Species abbreviations: NA, *Niviventer andersoni*; BB, *Berylmys bowersi*; RL, *Rattus losea*. Organ abbreviations: Lu, lung; Li, liver; Sp, spleen; Ki, kidney; In, intestine. This figure complements the workflow shown in Figure 2 by quantitatively illustrating read retention across processing steps.

A total of 303 million clean reads were generated from 15 pooled samples representing three rodent species. This sequencing depth allows exploratory RNA virome profiling but remains limited for comprehensive viral discovery, particularly for low-abundance viral taxa. Following stringent host decontamination and rRNA depletion, 1198 viral contigs were assembled and identified (N50 = 1517 bp). Taxonomic annotation revealed that these viral signatures belonged to 37 families, 14 genera, and 28 species. The abundance of viral reads across samples ranged from 0.27% to 5.25%, indicating that the host-depletion strategy effectively enriched the viral fraction (Figure 3).

### 3.2. Viral Community Composition and Tissue Tropism

**Figure 4:**
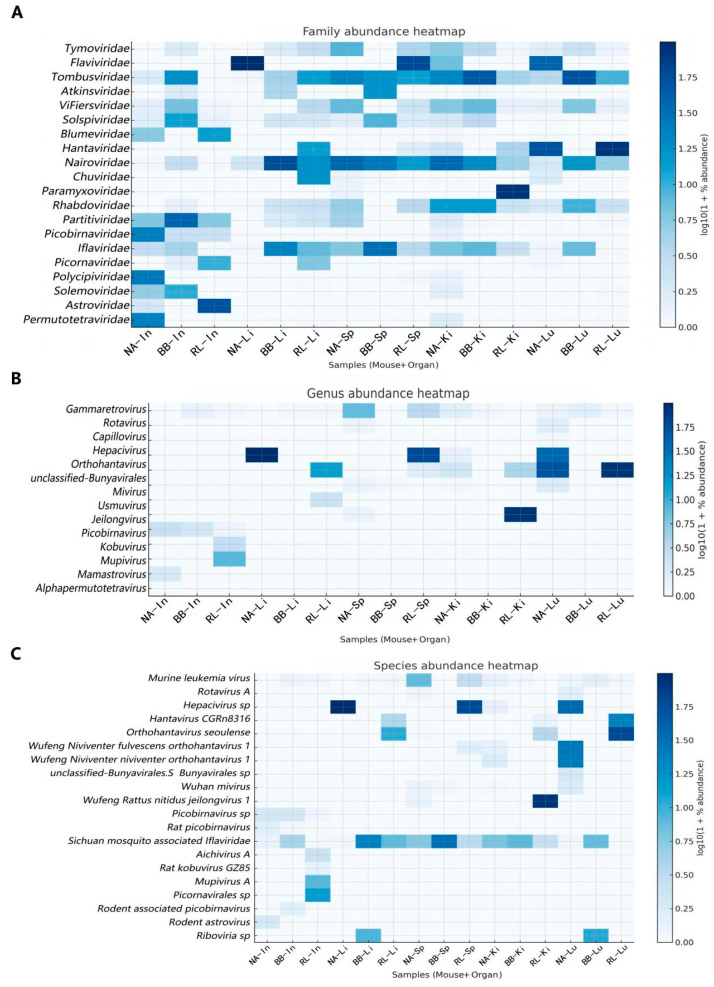
Heatmap profile of viral abundance and tissue tropism across three edible rodent species. (**A**) Family level heatmap showing the top 20 viral families ranked by total abundance. (**B**) Genus level heatmap showing all identified viral genera. (**C**) Species level heatmap showing the top 20 viral species ranked by total abundance. The heatmaps display the relative abundance of viral taxa across 15 pooled samples. The color gradient represents viral load normalized as log10-transformed reads per kilobase per million mapped reads (RPKM), with darker colors indicating higher abundance. Sample nomenclature: NA, *Niviventer andersoni*; BB, *Berylmys bowersi*; RL, *Rattus losea*; Lu, lung; Li, liver; Sp, spleen; Ki, kidney; In, intestine.

RPKM-based abundance profiling revealed host-associated differences and tissue-associated enrichment patterns in viral abundance (Figure 4).

#### 3.2.1. Virome of Parenchymal Organs

The liver, spleen, lungs, and kidneys were dominated by members of the families *Flaviviridae*, *Hantaviridae*, and *Paramyxoviridae*. Specifically, the genus *Hepacivirus* was highly abundant in the liver of *N. andersoni* (Sample Li1; RPKM = 98.32). The genus Orthohantavirus showed marked pulmonary enrichment, being predominantly detected in the lungs of *R. losea* and *N. andersoni*. Additionally, *Jeilongvirus* showed specific enrichment in the kidneys of *R. losea* (RL-Ki3; RPKM = 86.39).

#### 3.2.2. Virome of the Intestine

The intestinal virome displayed higher diversity compared to that in parenchymal organs. The gut of *R. losea* was dominated by *Astroviridae* (mainly *Kobuvirus*), whereas the intestines of *Berylmys bowersi* and *N. andersoni* were enriched with *Partitiviridae* and *Polycipiviridae*, respectively.

#### 3.2.3. Dietary-Associated Viral Signals

High abundances of *Iflaviridae* and *Tombusviridae* were detected in certain liver and spleen samples. These likely represent dietary residues reflecting the insectivorous and herbivorous feeding habits of the hosts, rather than active infections.

### 3.3. Viral Diversity and Drivers of Community Structure

#### 3.3.1. Host Identity Effect

**Figure 5:**
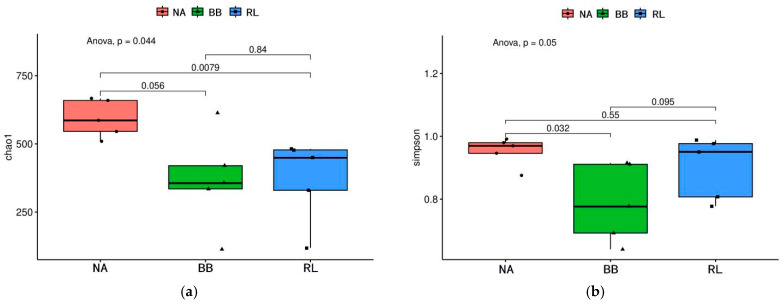

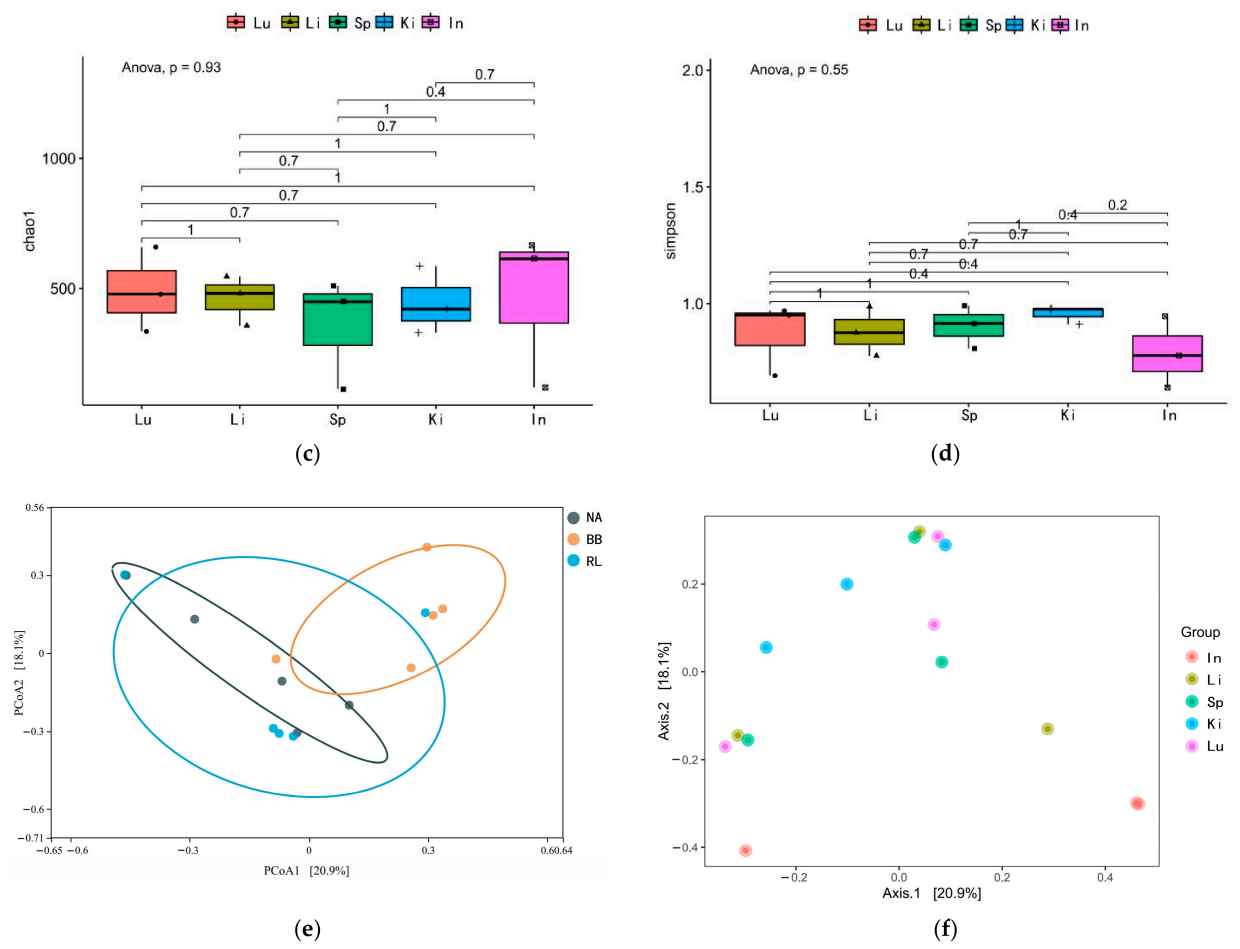
Alpha and beta diversity of viral communities across host species and organ types (**a**–**f**). Comparison of alpha diversity indices (Chao1 and Simpson) among three host species (**a**,**b**) and five organ types (**c**,**d**). (**e**,**f**) Principal coordinate analysis (PCoA) based on Bray–Curtis distances illustrating viral community structure clustered by host species (**e**) and organ type (**f**). Abbreviations: NA, *Niviventer andersoni*; BB, *Berylmys bowersi*; RL, *Rattus losea*; Li, liver; Sp, spleen; Lu, lung; Ki, kidney; In, intestine. Statistics: Statistical significance was evaluated using Kruskal–Wallis tests for alpha diversity and PERMANOVA for beta diversity.

Alpha diversity analysis showed significant differences in viral richness among host species. *N. andersoni* (NA) had the highest Chao1 index (mean = 593.2), significantly higher than that of *R. losea* (Kruskal–Wallis test, *p* = 0.0079). For beta diversity, Bray–Curtis-based PCoA showed that samples clustered primarily by host species, with the first two axes explaining 39.0% of the variation (Figure 5e). Although confidence ellipses partially overlapped, group centroids were distinct, with *B. bowersi* (BB) showing the tightest clustering.

#### 3.3.2. Organ Type Effect

In contrast to the host-driven pattern, Chao1 and Simpson indices did not differ significantly among organ groups (*p* > 0.05). PCoA showed extensive overlap among organ types, indicating no distinct organ-specific clustering at the community level (Figure 5f).

### 3.4. Molecular Evolutionary Characteristics of Key Zoonotic Viruses

To assess zoonotic potential, we performed phylogenetic analyses of the polymerase genes of two identified pathogens of significant public health concern: Seoul virus (SEOV) and Rotavirus A (RVA).

#### 3.4.1. Phylogenetic Analysis of Seoul Virus (SEOV)

**Figure 6:**
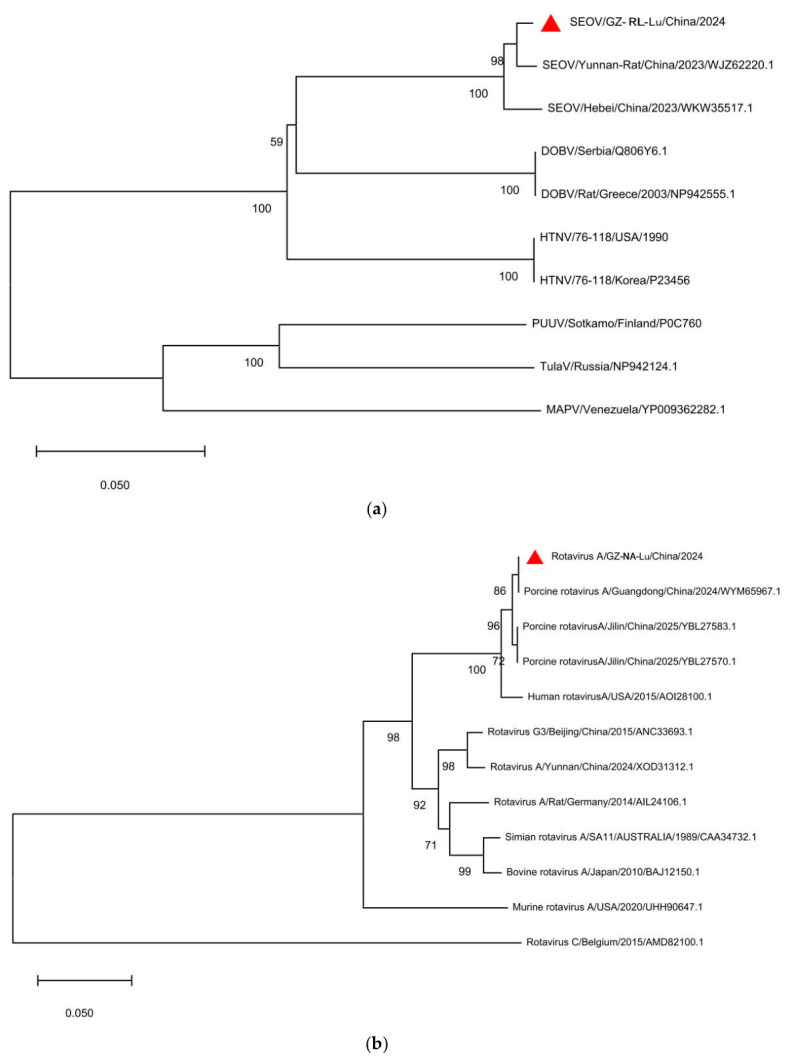
Phylogenetic trees based on viral RdRp amino acid sequences. (**a**) Phylogenetic tree of *Seoul virus* (SEOV), highlighting the evolutionary position of the strain detected in the RL-Lu sample (lung of *Rattus losea*; indicated by a red triangle). (**b**) Phylogenetic tree of Group A Rotavirus (RVA) based on VP1 proteins, showing that the strain detected in the NA-Lu sample (lung of *Niviventer andersoni*; indicated by a red triangle) clusters with porcine-origin strains. Methods: The phylogenetic trees were constructed using the maximum likelihood (ML) method. Bootstrap support values calculated from 1000 replicates are shown at the nodes (only values >70% are displayed).

The maximum likelihood (ML) tree based on the L segment-encoded RdRp amino acid sequences (Figure 6a) placed the sequence obtained from the RL lung sample (Strain ID: SEOV/GZ-RL-Lu) within the Seoul virus clade. Topologically, GZ-RL-Lu clustered at a terminal node with a 2023 isolate from Yunnan, China (SEOV/Yunnan- Rat/China/2023), supported by a bootstrap value of 98%. Sequence comparison showed that GZ-RL-Lu shared 98.93% nucleotide identity with this regional epidemic strain.

#### 3.4.2. Phylogenetic Analysis of Rotavirus A (RVA)

Phylogenetic analysis based on VP1 protein (RdRp) amino acid sequences placed the sequence from the NA lung sample (Strain ID: GZ-NA-Lu) within the Rotavirus A (RVA) evolutionary group (Figure 6b). Unlike typical murine RVA reference strains, GZ-NA-Lu clustered with a porcine RVA reference strain from Guangdong, China, forming an independent branch with 100% bootstrap support. The sequence shared 99.73% amino acid identity with this porcine-origin strain, suggesting a close phylogenetic relationship with porcine-associated RVA and a potential wildlife–livestock ecological link.

## 4. Discussion

This study provides the first systematic characterization of the multi-organ RNA virome of edible rodents in the Qiandongnan region. Beyond describing viral community composition, our findings offer insights into potential biosecurity concerns associated with local dietary practices.

Consistent with recent investigations into wildlife viromes [27,28], our data suggest that host identity may be an important determinant of viral community structure. *Niviventer andersoni* (NA) exhibited relatively high viral diversity and richness, which may be associated with its broader ecological range and synanthropic behavior—frequently inhabiting areas at the interface between human settlements and agricultural environments [29]. The observed overlap in viral composition between parenchymal organs and the gut suggests that certain viruses (e.g., Hepacivirus and *Jeilongvirus*) may be distributed across multiple tissues, potentially reflecting systemic exposure or transient circulation rather than confirmed systemic infection [30,31,32].

Hemorrhagic fever with renal syndrome (HFRS), caused by *Orthohantavirus*, remains a major public health concern in China. The detection of SEOV-related sequences in the lungs of *Rattus losea*, with high similarity to strains from neighboring regions, is consistent with the broader circulation of *hantaviruses* in China [33]. Because lungs are associated with viral shedding, handling and processing rodent tissues without protective measures may represent a potential exposure pathway. However, viral RNA detection alone does not confirm active infection or transmission; therefore, these findings should be interpreted as possible exposure signals rather than direct evidence of transmission.

A notable finding was the detection of sequences closely related to porcine Rotavirus A (RVA) in the lungs of *Niviventer andersoni*. Although rotaviruses generally show host specificity, cross-species transmission and reassortment have been reported, with pigs often acting as potential mixing hosts [34]. Given that NA frequently inhabits areas near farms and villages, these sequences may reflect environmental exposure or indirect contact at the wildlife–livestock interface rather than confirmed spillover. Although RVA is primarily enteric, extra-intestinal detection has been reported under certain conditions [35,36,37]. Thus, the lung signal may reflect non-specific viral RNA distribution, environmental contamination, or inhalation of virus-containing particles. Because rotaviruses have segmented genomes, reassortment analysis would require more complete genomic data, which were not available in this study.

Beyond SEOV and RVA, other viral groups identified in this study also warrant attention. The presence of *Hepacivirus* in the liver of *Niviventer andersoni* is consistent with previous evidence that rodents may harbor diverse hepaciviruses [38]. Although their direct pathogenic relevance to humans remains unclear, these findings highlight the diversity of rodent-associated viral populations. The detection of *Jeilongvirus* (*Paramyxoviridae*) in the kidneys of *Rattus losea* suggests possible viral shedding through urine. Given that some *paramyxoviruses* are associated with zoonotic infections [39], environmental contamination via excreta may represent a potential, though unconfirmed, exposure route. Overall, handling and consuming rodents, particularly internal organs, may provide opportunities for contact with viral agents; however, these risks should be interpreted as exposure potential rather than confirmed transmission pathways. Other viral groups were not subjected to detailed phylogenetic analysis because limited sequence completeness or insufficient reference data restricted robust evolutionary inference.

This study has several limitations. First, pooled samples increased sequencing depth but limited assessment of inter-individual variation and prevented accurate estimation of individual-level prevalence. Therefore, it remains unclear whether detected viral sequences were consistently present across animals or mainly contributed by a subset of individuals. Individual samples were not retained for parallel validation; future studies should incorporate individual-level sampling to validate these findings. Second, although 303 million clean reads were generated from 15 pooled samples, sequencing depth remains limited for comprehensive virome discovery in tissue metatranscriptomic datasets, where host-derived reads are abundant. Low-abundance or highly divergent viral sequences may therefore have been missed. Third, viral detection relied on metatranscriptomic sequencing alone, without PCR, Sanger sequencing, viral isolation, or infectivity assays. Therefore, the detected viral sequences should be interpreted as sequence-based evidence rather than confirmed viral presence. Finally, sampling was restricted to a single season and location, and phylogenetic analyses were limited to selected conserved genes. Future studies incorporating individual-level sampling or biological replicate pools, deeper sequencing, quantitative validation, viral isolation, and broader spatiotemporal sampling are needed to clarify viral prevalence, tissue distribution, and public health relevance.

## 5. Conclusions

In conclusion, this study provides a baseline characterization of the multi-organ RNA virome of edible rodents in Southwest China and reveals the presence of diverse viral signatures at the sequence level, including those related to zoonotic and livestock-associated viruses. While these findings do not demonstrate active infection or confirmed cross-species transmission, they suggest a potential interface for viral exchange between wildlife, livestock, and humans. The detection of viral sequences in tissues relevant to food handling underscores the importance of considering traditional dietary practices as potential pathways for pathogen exposure. These results highlight the need for continued surveillance and risk assessment within a One Health framework, particularly in regions where close interactions between wildlife, livestock, and humans are common.

## Data Availability

The sequencing data generated in this study have been deposited in the NCBI Sequence Read Archive (SRA) under BioProject accession number PRJNA1458619 and will be released upon publication. The assembled viral genome sequences have been deposited in GenBank under accession numbers PZ350881 (SEOV/GZ-RL-Lu/China/2024) and PZ350882 (Rotavirus_A/GZ-NA-Lu/China/2024), which will be publicly available upon publication.

## Abbreviations

The following abbreviations are used in this manuscript:

NA	*Niviventer andersoni*
BB	*Berylmys bowersi*
RL	*Rattus losea*
Lu	Lung
Li	Liver
Sp	Spleen
Ki	Kidney
In	Intestine
PE150	Paired-end sequencing with 150 bp reads
RPKM	Reads per kilobase per million mapped reads.
